# Dermatoscopy for the rapid diagnosis of Talaromyces marneffei infection: a case report

**DOI:** 10.1186/s12879-019-4351-2

**Published:** 2019-08-09

**Authors:** Jiayi Xian, Xiaowen Huang, Qiaofei Li, Xiaoming Peng, Xuebiao Peng

**Affiliations:** Department of Dermatology, Nanfang Hospital, Southern Medical University, Guangzhou, 510515 China

**Keywords:** Talaromyces marneffei, Dermatoscopy, Rapid diagnosis

## Abstract

**Background:**

Talaromyces marneffei is a thermally dimorphic fungus endemic in south-east Asia. It predominantly occurs in both immunocompromised and immunosuppressed patients and can be fatal if diagnosis and treatment are delayed. The clinical manifestations of T. marneffei infection are nonspecific and rapid diagnosis of T. marneffei infection remains challenging.

**Case presentation:**

A 24-year-old man came to our outpatient department with the sign of common skin lesions. The lesions were cuticolor follicular papules with or without central umbilication, nodules and acne-like lesions, which are common in syringoma, steatocystoma multiplex and trichoepithelioma. A dermatoscopy examination was performed to differentiate these skin lesions. The dermatoscopic images revealed circular or quasi-circular whitish amorphous structure with a central brownish keratin plug, providing the diagnostic clues of T. marneffei infection. Therefore, a skin scrapings culture, skin biopsy and serological detection for human immunodeficiency virus (HIV) were performed. The final diagnosis of this patient was T. marneffei and HIV co-infection.

**Conclusion:**

Rapid diagnosis of T. marneffei infection is clinically challenging since presenting clinical manifestations are nonspecific with significant overlap with other common conditions. This case highlights that dermatoscopy is a promising tool for the rapid diagnosis of T. marneffei infection in patients with nonspecific skin lesions, assisting clinicians to avoid delayed diagnosis or misdiagnosis.

## Background

Talaromyces marneffei, formerly called *Penicillium marneffei*, can cause severe and disseminated infection in immunocompromised individuals, especially in those who have HIV infection. Early diagnosis of T. marneffei infection is difficult because presenting clinical manifestations are nonspecific with significant overlap with other common conditions. A previous clinical study revealed that the delayed diagnosis of T. marneffei infection is an independent predictor for the early mortality [1]. There is still a need for more methods to diagnose T. marneffei infection rapidly, especially for those patients with nonspecific manifestations.

Dermatoscopy is a non-invasive diagnostic technology providing rapid observation of epidermal and dermal morphological features which are imperceptible to our naked eyes. According to previous studies, dermatoscopy has been demonstrated to be useful in various kinds of dermatoses, including cutaneous neoplasms, ectoparasitic infestations, hair and nail disorders, cutaneous/mucosal diseases, psoriasis and other dermatoses [2, 3]. Nowadays, dermatoscopy can be utilized to differentiate cutaneous disorders, assist clinical diagnosis and evaluate prognostic, acting as a useful and relatively simple examination in outpatient department. But up to now, using dermatoscopy to assist in diagnosing T. marneffei infection is rarely reported.

Herein, we present a 24-year-old male patient with nonspecific skin lesions who was suspected of syringoma based on his clinical symptom. However, he was presumptively diagnosed as T. marneffei infection within minutes after dermatoscopy examination. The diagnosis of T. marneffei infection was confirmed by fungal culture and histopathological examination a few days later.

## Case presentation

**Fig. 1:**
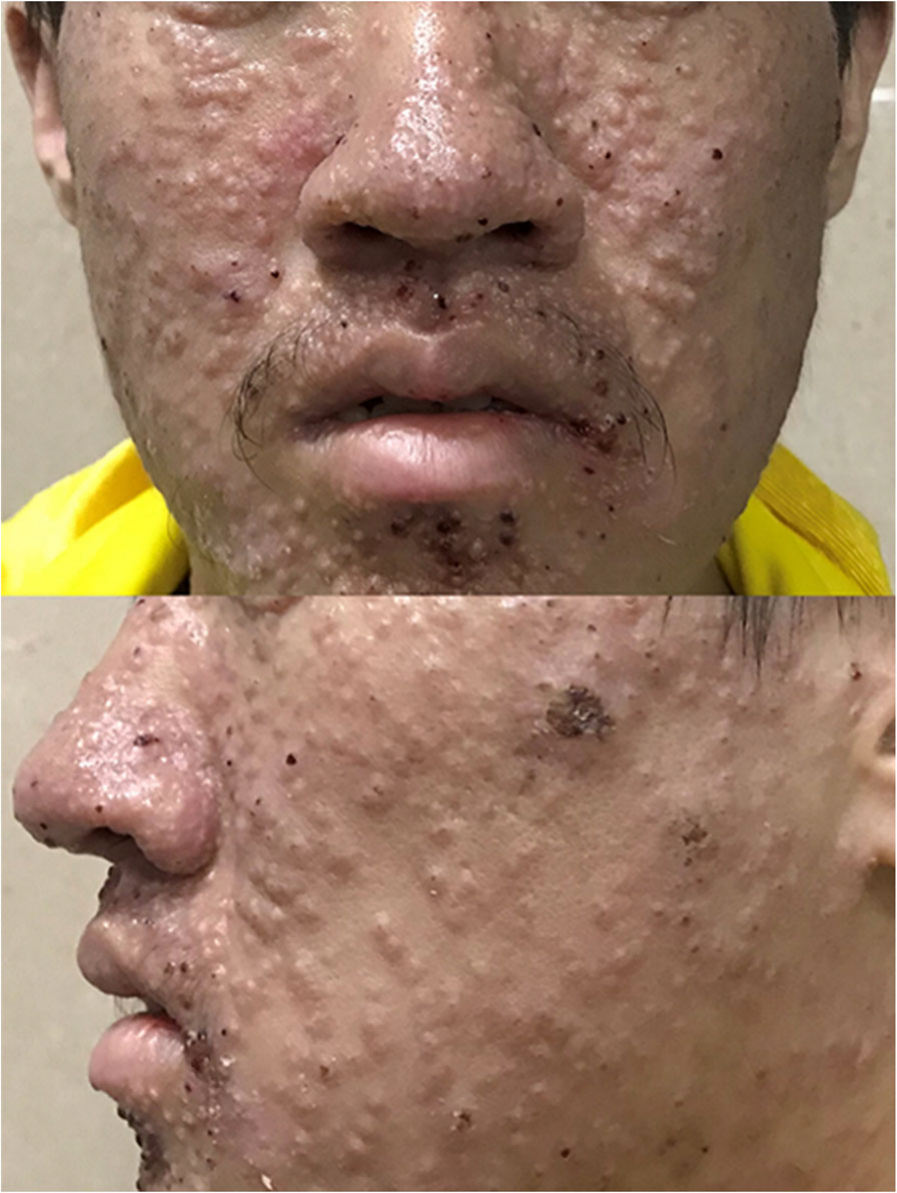
Physical examination. Multiple cuticolor follicular papules with or without central umbilication, nodules and acne-like lesions were developed densely on his face, neck and upper anterior chest. Some of them were covered with reddish brown crusts

**Fig. 2:**
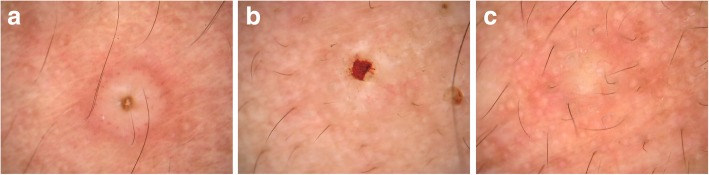
Three dermatoscopic patterns of 17 papules. **a** Circular or quasi-circular whitish amorphous structure with a central brownish keratin plug; (**b**) irregular whitish amorphous structure with hemorrhagic area of reddish brown color; (**c**) irregular homogeneous whitish amorphous structure

A 24-year-old man came to our dermatology outpatient office with a 1-week history of asymptomatic skin lesions which had developed densely on his face, neck and upper anterior chest. Physical examination revealed multiple cuticolor follicular papules with or without central umbilication, nodules and acne-like lesions. Some of them were covered with reddish brown crusts (Fig. 1). Based on his clinical symptom, the presumptive diagnosis of this patient was syringoma. Differential diagnoses including steatocystoma multiplex and trichoepithelioma were also under consideration. The dermatoscopic features of these diseases have been demonstrated to be different [4–6]. Therefore, dermatoscopy examination has the potential to help in diagnosing this patient. A dermatoscopy examination was performed randomly on 17 papules on his face. Surprisingly, the dermatoscopic images of twelve (12/17, 70.6%) papules showed circular or quasi-circular whitish amorphous structure with a central brownish keratin plug (Fig. 2a). This dermatoscopic pattern is the typical“white jade coin pendant” sign which we have observed in another patient of T. marneffei infection before [7]. The dermatoscopic characteristics of four (4/17, 23.5%) papules showed irregular whitish amorphous structure with hemorrhagic area of reddish brown color (Fig. 2b). One papule (1/17, 5.9%) presented an irregular homogeneous whitish amorphous structure (Fig. 2c). Because the dermatoscopic images provided the diagnostic clues of T. marneffei infection, further relevant examination was performed.

**Fig. 3:**
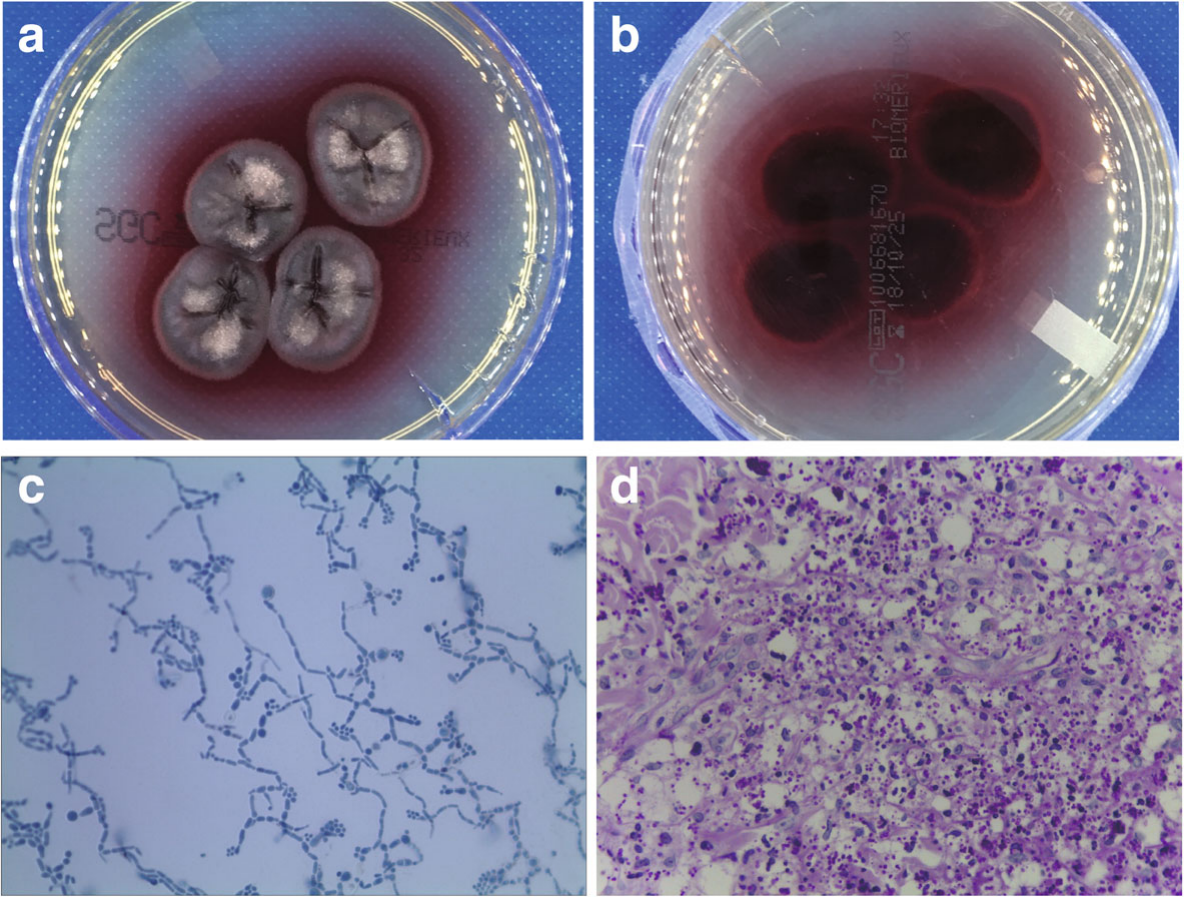
Mycological and histopathology findings. **a** A colony was producing a red-wine colored pigment that diffused into dextrose agar plate incubated; (**b**) Reverse side of the colony; (**c**) Staining with lactophenol cotton blue revealed the hyphae were highly branched and these branched hyphae had already undergone arthroconidiogenesis; (**d**) Histopathological examination revealed abundant yeast-like organisms in the cytoplasm of histocytes. The organisms were spherical to oval, about 3–8 um in diameter and occasional contained septum. (periodic acid-Schiff staining, × 400)

A few days later, the skin scrapings culture grew fungi. The fungi grew as fluffy whitish colonies (Fig. 3a) that produced red-wine colored diffusible pigment on Sabouraud dextrose agar (Fig. 3b). Staining with lactophenol cotton blue revealed the hyphae were highly branched and these branched hyphae had already undergone arthroconidiogenesis (Fig. 3c). Histopathology of a skin biopsy sampling from a papule on upper anterior chest revealed abundant yeast-like organisms in the cytoplasm of histocytes by periodic acid-Schiff (PAS) stain. The organisms were spherical to oval, about 3–8 um in diameter and occasional contained septum (Fig. 3d). For serological detection of HIV, the enzyme-linked immunosorbent assay (ELISA) was used as screening test followed by Western blot for confirmation. The patient’s blood specimen was repeatably reactive by ELISA and was also positive by Western blot for anti-HIV-1 antibody. The final diagnosis of this patient was T. marneffei and HIV co-infection.

After the 2-week treatment with intravenous amphotericin B and antiretroviral therapy (ART), the patient’s condition improved. Thus, he was discharged with oral itraconazole (200 mg twice daily). During 3-month follow-up, his cutaneous lesions were substantially improved, while the oral itraconazole were continuously used to prevent relapses of T. marneffei infection.

## Discussion and conclusions

T. marneffei infection is incrementally reported in both immunocompromised and immunosuppressed patients. In recent years, it has been observed even in immunocompetent patients [8]. Annually, about 50,000 acquired immunodeficiency syndrome (AIDS) patients are newly infected by T. marneffei, resulting in approximately 10% mortality in Southeast Asia [9]. The clinical presentations of T. marneffei infection are nonspecific, including fever, respiratory symptoms, lymphadenopathy, gastrointestinal complains, skin lesions, hepatomegaly, and/or splenomegaly [1, 10]. The nonspecific skin lesions are observed in 71% of patients with T. marneffei infection [11]. Similarity of these manifestations to those observed in other diseases may lead to a delayed diagnosis or erroneous diagnosis.

Nowadays, definitive diagnosis of T. marneffei infection is generally based on fungal culture. Previous studies have showed high sensitivities from skin scrapings (95%), lymph node (85%) and blood (84%) to diagnose T. marneffei infection [10].

There are other methods to make the presumptive diagnosis of T. marneffei infection, including microscopic examination of the Wright’s-stained samples of bone-marrow aspirate and touch smear samples of the skin-biopsy or lymph-node biopsy [11]. Recently, peripheral blood smear [12] and high-throughput sequencing of multifarious specimens [13] were reported to assist the rapid diagnosis of T. marneffei infection.

Considering that these are all invasive tests, it’s difficult to perform as routine clinical practice. There has been increasing interest in the use of serodiagnostics methods, including the immunodiffusion (ID) test, the indirect fluorescent-antibody test, the ELISA-based antibody test and so on [14]. However, for the reason that these patients are immunocompromised, the titres of their antibodies may be low, which may influence the serodiagnostics result. More studies are needed to verify their actual specificity, sensitivity and validity. Therefore, there is still a need for more methods to diagnose T. marneffei infection rapidly, especially for those patients with nonspecific manifestations.

Dermatoscopy, a noninvasive diagnostic technology, can make the microcosmic morphological features become visible to our unaided eyes, improving our knowledge about the morphology of numerous kinds of skin lesions. Previous studies have demonstrated that it can improve the diagnostic accuracy for both pigmented and nonpigmented lesions in comparison with examination with the naked eyes [15, 16]**.** Nowadays, dermatoscopy is increasingly conducted by clinicians to inform morphological features of skin lesions, representing a practical and relatively simple technology in daily clinical practice. But up to now, few studies have reported using dermatoscope to assist diagnosing T. marneffei infection. We previously analyzed four patterns of the dermatoscopic features of disseminated talaromycosis marneffei (DTM), describing the “white jade coin pendant” sign, the whitish amorphous structure with a central plug or hemorrhage, as the dermatoscopic characteristic of DTM [7].

As observed in this case, the patient came to our outpatient clinic with the only symptom of skin lesions, which was nonspecific in physical examination. However, the emblematic “white jade coin pendant” dermatoscopic feature gave some sort of diagnostic clues about T. marneffei infection, leading to more relevant examinations further. Finally, the culture medium grew T. marneffei and histopathological examination with PAS staining was positive, confirming the presumptive diagnosis of T. marneffei infection ahead. This case highlights that dermatoscopy has the utility for the expeditious diagnosis of T. marneffei infection.

There were three kinds of dermatoscopic features we discovered in this case, which are considered to be associated with different developmental stages of skin lesions. In the early stage, T. marneffei infection shows a homogeneous whitish amorphous structure without any other characteristics, while it can develop hemorrhagic ulceration or a central keratin plug in the fully-developed stage [7].

The mainstay of standard therapy is 2 weeks of intravenous amphoteracin B, followed by 10 weeks of oral itraconazole. A secondary prophylaxis with oral itraconazole is recommended, as it was demonstrated that it can prevent relapses of T. marneffei infection [17]. It is also recommended that it can be discontinuated until there has been significant immune restoration from highly active antiretroviral therapy (HAART).

Rapid diagnosis of T. marneffei infection is clinically challenging since presenting clinical manifestations are nonspecific with significant overlap with other common conditions. The evaluation of patients with these manifestations can be complex and fraught with diagnostic pitfalls. Our case suggests when treating patients with nonspecific skin lesions, clinicians should be aware of the possibility of T. marneffei infection and take the dermatoscopy examination into account early during the diagnostic process. Vigilance is essential to identify skin lesions that masquerade as common dermatoses that may lead to erroneous diagnosis. This case highlights that dermatoscopy is a promising tool for the rapid diagnosis of T. marneffei infection in patients with nonspecific skin lesions, assisting clinicians to avoid delayed diagnosis or misdiagnosis.

## Data Availability

All data generated or analyzed during this study are included in this published article.

## Abbreviations

AIDS: Acquired immunodeficiency syndrome
ART: Antiretroviral therapy
DTM: Disseminated talaromycosis marneffei
ELISA: Enzyme-linked immunosorbent assay
HAART: Highly active antiretroviral therapy
HIV: Human immunodeficiency virus
ID: Immunodiffusion
PAS: Periodic acid-Schiff

## References

[1] Zheng J, Gui X, Cao Q, Yang R, Yan Y, Deng L, Lio J (2015). A clinical study of acquired immunodeficiency syndrome associated Penicillium Marneffei infection from a non-endemic area in China. PLoS One.

[2] Yelamos O, Braun RP, Liopyris K, Wolner ZJ, Kerl K, Gerami P, Marghoob AA (2019). Dermoscopy and dermatopathology correlates of cutaneous neoplasms. J Am Acad Dermatol.

[3] Micali G, Lacarrubba F, Massimino D, Schwartz RA (2011). Dermatoscopy. Alternative uses in daily clinical practice. J Am Acad Dermatol.

[4] Sakiyama M, Maeda M, Fujimoto N, Satoh T (2014). Eruptive syringoma localized in intertriginous areas. J Dtsch Dermatol Ges.

[5] Navarrete-Dechent C, Bajaj S, Marghoob AA, Gonzalez S, Munoz D (2016). Multiple familial trichoepithelioma: confirmation via dermoscopy. Dermatol Pract Concept.

[6] Sharma A, Agrawal S, Dhurat R, Shukla D, Vishwanath T (2018). An unusual case of facial Steatocystoma multiplex: a Clinicopathologic and Dermoscopic report. Dermatopathology.

[7] Li Q, Wang C, Zeng K, Peng X, Wang F (2018). AIDS-associated disseminated talaromycosis (penicilliosis) marneffei. J Dtsch Dermatol Ges.

[8] Wang P, Chen Y, Xu H, Ding L, Wu Z, Xu Z, Wang K (2017). Acute disseminated Talaromyces marneffei in an immunocompetent patient. MYCOPATHOLOGIA.

[9] Armstrong-James D, Meintjes G, Brown GD (2014). A neglected epidemic: fungal infections in HIV/AIDS. Trends Microbiol.

[10] Le T, Wolbers M, Chi NH, Quang VM, Chinh NT, Huong Lan NP, Lam PS, Kozal MJ, Shikuma CM, Day JN (2011). Epidemiology, seasonality, and predictors of outcome of AIDS-associated Penicillium marneffei infection in Ho Chi Minh City, Viet Nam. Clin Infect Dis.

[11] Supparatpinyo K, Khamwan C, Baosoung V, Sirisanthana T, Nelson KE (1994). Disseminated Penicillium marneffei infection in Southeast Asia. Lancet.

[12] Othman J, Brown CM (2018). Talaromyces marneffei and dysplastic neutrophils on blood smear in newly diagnosed HIV. BLOOD.

[13] Zhu Y, Ai J, Xu B, Cui P, Cheng Q, Wu H, Qian Y, Zhang H, Zhou X, Xing L, et al. Rapid and precise diagnosis of disseminated T.marneffei infection assisted by high-throughput sequencing of multifarious specimens in a HIV-negative patient: a case report. BMC Infect Dis. 2018;18(1). 10.1186/s12879-018-3276-5 PMC6081951 30086724

[14] Vanittanakom N, Cooper CJ, Fisher MC, Sirisanthana T (2006). Penicillium marneffei infection and recent advances in the epidemiology and molecular biology aspects. Clin Microbiol Rev.

[15] Sinz C, Tschandl P, Rosendahl C, Akay BN, Argenziano G, Blum A, Braun RP, Cabo H, Gourhant J, Kreusch J (2017). Accuracy of dermatoscopy for the diagnosis of nonpigmented cancers of the skin. J Am Acad Dermatol.

[16] Kittler H, Pehamberger H, Wolff K, Binder M (2002). Diagnostic accuracy of dermoscopy. LANCET ONCOL.

[17] Supparatpinyo K, Perriens J, Nelson KE, Sirisanthana T (1998). A controlled trial of itraconazole to prevent relapse of Penicillium marneffei infection in patients infected with the human immunodeficiency virus. N Engl J Med.

